# Self-reported psychological problems and coping strategies: a web-based study in Peruvian population during COVID-19 pandemic

**DOI:** 10.1186/s12888-021-03326-8

**Published:** 2021-07-13

**Authors:** Rita J. Ames-Guerrero, Victoria A. Barreda-Parra, Julio C. Huamani-Cahua, Jane Banaszak-Holl

**Affiliations:** 1 School of Psychology, Universidad Católica de Santa María, Arequipa, Peru; 2 School of Psychology, Universidad Nacional de San Agustín de Arequipa, Arequipa, Peru; 3 School of Psychology, Universidad Católica San Pablo, Arequipa, Peru; 4 School of Public Health and Preventive Medicine, Monash University, Melbourne, Australia

**Keywords:** Mental health, Psychological disturbances, Coping strategies, Public health, Primary prevention

## Abstract

**Background:**

The Coronavirus pandemic has disrupted health systems across the world and led to major shifts in individual behavior by forcing people into isolation in home settings. Its rapid spread has overwhelmed populations in all corners of Latin-American countries resulting in individual psychological reactions that may aggravate the health crisis. This study reports on demographics, self-reported psychological disturbances and associated coping styles during the COVID-19 pandemic for the Peruvian population.

**Methods:**

This cross-sectional study uses an online survey with snowball sampling that was conducted after the state of emergency was declared in Perú (on April 2nd). The General Health Questionnaire (GHQ-28) was used to identify somatic symptoms, incidence of anxiety/ insomnia, social dysfunction and depression and the Coping Strategy Questionnaire (COPE-28) mapped personal strategies to address recent stress.

**Results:**

434 self-selected participants ranging in age from 18 to 68 years old (Mean age = 33.87) completed the survey. The majority of participants were women (61.30%), aged between 18 and 28 (41.70%), well-educated (> = 85.00%), Peruvian (94.20%), employed (57.40%) and single (71.20%). 40.8% reported psychological distress, expressing fear of coronavirus infection (71.43%). Regression analysis shows that men had lower somatic-related symptom (β = − 1.87, 95%, CI: − 2.75 to −.99) and anxiety/insomnia symptom (β = − 1.91, 95% CI: − 2.98 to 0.84) compared to women. The risk for depression and social dysfunction are less likely with increasing age. Educational status was protective against developing psychological conditions (*p* < 0.05). While active responses (acceptance and social support) are scarcely used by individuals with psychological distress; passive strategies (such as denial, self-distraction, self-blame, disconnection, and venting) are more commonly reported.

**Conclusion:**

This study provides a better understanding of the psychological health impact occurring during the COVID-19 pandemic on the Peruvian population. About half of the respondents reported psychological distress and poor coping responses. This evidence informs the need for broader promotional health policies focused on strengthening individual’s active strategies aiming at improving emotional health and preventing psychiatric conditions, during and after the COVID-19 pandemic.

## Background

To date (June 10th, 2020), more than 400,000 deaths worldwide have been attributed to the coronavirus [1]. Despite the deployment of several public health strategies to prevent continued transmission of the virus at the global level [1–3], the subjective perception of risk within the population represents a latent threat that may potentially trigger a wide variety of individual behavioral and emotional responses. Therefore, during this pandemic complex disturbances are highly likely to occur [4–6] and recent research has found a significant association between the current COVID-19 pandemic and the emergence of mental disturbances [7–9].

Early reports from multiple studies during the epidemic phase in China confirm moderate to severe psychological impact, described as severe states of distress and deteriorated psychological health [10–13]. In a cross-sectional nation-wide study, with nearly ﻿52,730 participants, psychological distress was identified among the one-third of the sample (35%) [11]. Another study, performed in Australia, showed that, mental conditions have worsened since the onset of quarantine. In this study, 5070 randomly selected participants were taken from the general population. About 78% of the respondents self-reported that their mental health was adversely compromised by symptoms of depression (62%), anxiety (50%) and stress (64%). Mostly vulnerable groups such as the unemployed, students, retired, and stay-at-home parents stated those symptoms [14]. The emergence of patterns of emotional distress [15], anxiety, depression, sleep difficulties [8–10], and an increase in risk behaviors, such as substance use and smoking [14, 16], have now been suggested. As financial instability resulting from job loss and massive social isolation became more prevalent, these clinical conditions might intensify their status [16].

Considering that coping mechanisms are unpredictable during stressful life events [17], a strong link has been demonstrated between physical health, psychological well-being, and the use of active coping styles [18]. Many experts documented pandemics and its impact on the psychological health [19, 20], those reports essentially provide considerable reasons for assessing human behavior when it comes to cladding life-threatening circumstances. For instance, Li et al. traced the emotional and cognitive responses of Chinese population during the COVID-19 outbreak through a social network dubbed Weibo. This recent study revealed an intensification of negative emotions (anxiety, depression and indignation) and coping behaviors mostly related to increased leisure activity and religion-based responses [9]. In this context, the starting point of our research is the conceptual analysis given by Lazarus, who explains the stress mechanism suggesting two types of responses [17]. While some individuals may proactively explore options for assistance, others remain in the space of their own loneliness, worsening the burden of their illness. Either passive or active behavior-related strategies would predict the evolution of disease symptoms. Thus, there is a critical demand to identify coping mechanisms to reduce the risk of coronavirus spreading within the population.

Global pandemics tend to create confusion, sense of urgency, fear and perplexity, which may threaten emotional stability of entire families [21–23]. What is worth noting from prior reports is that, the behavioral mechanisms remain unclear to prevent people from reaching states of “collective hysteria” [24]. The unknown nature of the coronavirus disease may lead to an increased in perceived health vulnerability [25] and the possible emergence of mental disturbances in general population, along with unadjusted behaviors, as suggested from previous pandemics [26]. Few researchers have addressed the individual perception during global crisis. Concerns have arisen on how the ongoing pandemic may influence psychological health in the general population. It is worth mentioning the limited studies capturing the perspective directly from the affected population, mental health-related research has been neglected particularly in the Latin American context. Given the increased exposure to develop disease [23, 27, 28] within the poorest and most vulnerable groups in society [10], exploring the coping mechanisms has become a central issue in enforcing behavioral awareness and monitoring. Thus, there is an urgent need to better understand mental health disruptions caused by unexpected events such as the COVID-19 pandemic in the Spanish-speaking community.

Particularly, Peru has been considerably affected by the COVID-19 pandemic, causing significant national alarm [29]. Although exposure to the virus outbreak has already been shown to be related to adverse physical effects, personal coping mechanisms to manage stress and identify psychological symptoms remain to be discussed [17, 30]. Therefore, the aim of our research was to broaden current knowledge about active or passive coping strategies and how they are linked to psychological health in the general population exposed to the COVID-19 pandemic in Peru.

## Methods

### Participants and procedures

Data from 434 individuals of the general population living in Peru were used. Furthermore, to determine the estimated sampling size, G-power statistic were used with a confidence interval of 0.10 and error range of 15%. We selected a cross-sectional survey design to examine the population’s psychological responses during the COVID-19 pandemic.

### Procedure

Given the pandemic, public government restricted physical interaction, then anonymous online forms were disseminated to likely participants through Peruvian, health, and wellbeing- related social networks (“salud y bienestar Peru”, “Ministerio de salud Peru”, “Comunidad de salud”, Peru) using a snowball sampling strategy. Participants completed the survey on a voluntary basis via their smartphones or desktops during March–April. Completing the survey took about 40 min. To recruit individuals, researchers considered only people living in Peru, able to provide informed consent (≥18 years), no monetary compensation was given for completing the questionnaire. The study protocol was approved by the ethics committee of “Catolica de Santa Maria” University *(ref. no. 167–2020).* The instruments were considered valid when fully completed; participants under 18 years old and those whose responses were biased by acquiescence or social desirability were excluded based on the questionnaire’s protocol.

#### General health questionnaire

The survey comprised the ***General Health Questionnaire (GHQ-28),*** a population-based, self-administered tool for screening mental disorders. Participants were asked about symptoms and/or discomfort they had experienced recently (in recent weeks) during the coronavirus pandemic. Each item is scored based on a 4-point scale containing 4 subscales of depression, anxiety, social dysfunction, and physical symptoms, ranging from “better/healthier than normal” option, through a “same as usual” and a “worse/more than usual” to a “much worse/more than usual” option [31].

The instrument has already been validated in spanish-speaking countries with adaptations for Latin American countries [32], alongside being reported in multiple Spanish studies [33, 34]. Reliability analysis by internal consistency was determined for this study by using the Mc Donald’s Omega Coefficient [35] with acceptable values for the depression subscale (ω = 0.83; IC95% = 0.81–0.85); anxiety/insomnia subscale (ω = 0.90; IC95% = 0.89–0.92); social dysfunction subscale (ω = 0.79; IC95% = 0.76–0.82) and the physical symptoms subscale (ω = 0.89; IC95% = 0.87–0.90). The presence of psychological disturbance was identified with a cut-off point 23/24 [36].

#### Coping strategies questionnaire

The survey also included the ***Coping strategies questionnaire (COPE-28), its*** brief version of 28 items measures behaviors and cognitive responses to stressors related to COVID-19. We used the Spanish version [37], which has 14 subscales (asking about self-distraction, active coping, denial, substance use, use of emotional support, use of instrumental support, behavioural disengagement, venting, positive reframing, planning, humor, acceptance, religion, self-blame). The tool identified active and passive strategies asking 4-point values (1: “I haven’t been doing this at all”; 4 “I’ve been doing this a lot”) [38].

Greater values indicate higher strategies to deal with stress. The COPE − 28 has been shown to have good validity and reliability in many Spanish studies [13, 37] and has been validated in Peru [39, 40]. In our sample, the questionnaire had good reliability, it was determined by using the Mc Donald Omega’s model ω = 0.858 (IC = 95%, 0.838–0.876) indicating high internal consistency for active coping subscales (ω = 0.714; IC95% = 0.67–0.75 and passive coping subscales (ω = 0.74; IC95% = 0.71–0.78).

### Data analysis

Respondents were asked to identify categorically key demographic information, which is subsequently analyzed using *Stata Statistical Software 15.0* for Windows [41] as proportions. Linear regression was used to calculate whether there were univariate associations between sociodemographic data, the GHQ scale and COPE-28 questionnaire.

To establish the relationship between psychological health and both active and passive coping strategies, structural equation models (SEM) were used. Path analysis [42] was done with comparative adjustment index (CAI), with values ≥ .90 [43] the root mean square error of approach (RMSEA), with values ≤ .80 [44] and the goodness-of-fit Index (GFI > .8 or > .9) was used to evaluate how well the models fit [45]. From the correlations, the final model was elaborated with path analysis, using data from participants scoring higher in psychological distress to determine the most used active and passive coping strategies within this group.

## Results

### Participant characteristics **(**Table 1**)**

From the 450 respondents who completed the survey, only 434 (38.70% males and 61.30% females) were recruited into the study with a response rate of 100% (Table 1). The mean age of participants was 33.87 ﻿ ± 12.6, whose age range from 18 to 68 years old coming from 16 departments of Perú. The majority were well-educated (> = 85.00%), Peruvian (94.20%), employed (57.40%) and single (71.20%). Regarding social factors, a great number were afraid of contracting coronavirus (71.40%), 47.70% were worried about limited access to cleaning products, and 38.90% about social distancing, followed by 27.80% worried about not being able to work. Regarding employment, 42,60% were unemployed at the quarantine period, and 40.09% were university students.

**Table 1:** Association between socio-demographic variables and indicators of general health status at covid-19

Variables	N(%)	Somatic symptom			Anxiety/Insomnia			Social Dysfunction			Severe Depression		
		Squared R^2^	Adjusted R-Squared(AR^2^)	*P*-value (95% CI)	R^2^	(AR^2^)	P-value (95% CI)	R^2^	(AR^2^)	P-value (95% CI)	R^2^	(AR^2^)	P-value (95% CI)
Gender													
Woman	266 (61.3)	0.039	0.037	Reference	0.028	0.026	Reference	0.001	− 0.002	Reference	0.005	0.005	Reference
Men	168 (38.7)			0.00*(− 2.75 to − .99) **β = − 1.87**			0.00*(− 2.98 to 0.84) **β = − 1.91**			0.59 (− 1.04 to 0.59) β = − 0.22			0.15 (− 1.32 to 0.20) β = − 0.56
Age													
18–28	181 (41.7)			Reference			Reference			Reference			Reference
29–38	124 (28.6)	0.013	0.004	−0.34 (− 1.57 to 0.54) β = − 0.51	0.022	0.013	0.02* (− 2.73 to − 0.19) **β = − 1.46**	0.034	0.025	0.05*(− 1.89 to 0.01) β = − 0.94			0.00*(− 2.21 to − 0.43) **β = − 1.32**
39–48	51 (11.8)			0.94 (− 1.39 to 1.49) β = 0.05			0.45 (− 1.07 to 2.39) β = 0.66			0.23 (− 2.08 to 0.50) β = − 0.79	0.042	0.033	0.04*(− 2.47 to − 0.06) **β = − 1.26**
49–58	54 (12.4)			0.74 (− 1.17 to 1.64) β = 0.23			0.24 (− 2.70 to 0.67) β = − 1.02			0.03*(− 2.61 to − 0.08) **β = − 1.35**			0.00*(− 3.10 to − 0.74) **β = − 1.92**
59–68	24 (5.5)			0.04*(− 4.06 to − 0.13) β = − 2.09			0.08 (− 4.45 to 0.28) β = − 2.08			0.00*(− 4.88 to − 1.34) **β = − 3.11**			0.00*(− 4.01 to − 0.70) **β = − 2.36**
Education level													
High school	64 (14.75)			Reference			Reference			Reference			Reference
College	174 (40.09)	0.876	**0.875**	0.001*(2.55 to 3.48) **β = 3.02**	0.502	0.489	0.006*(0.47 to 2.74) **β = 1.61**	0.312	0.307	0.001*(1.01 to 3.01) **β = 2.01**	0.236	0.231	0.001*(1.01 to 3.01) **β = 0.16**
Bachelor	116 (26.73)			0.001*(6.83 to 7.82) **β = 7.32**			0.001*(5.24 to 7.66) **β = 6.45**			0.001*(3.88 to 6.01) **β = 4.94**			0.001*(3.88 to 6.01) **β = 3.02**
Postgraduate	80 (18.43)			0.001*(12.6 to 13.7) **β = 13.16**			0.001*(9.87 to 12.5) **β = 13.18**			0.001*(5.82 to 8.13) **β = 6.98**			0.001*(5.82 to 8.13) **β = 4.84**
Nationality													
Peruvian	409 (94.2)	0.001	−0.001	Reference	0.001	−0.002	Reference	0.000	− 0.002	Reference	0.007	0.005	Reference
Foreign	25 (5.8)			0.51(−1.24 to 2.50) β = 0.63			0.66 (− 1.75 to 2.78) β = 0.52			0.67 (− 1.34 to 2.06) β = 0.36			0.07 (−0.13 to 3.05 β = 1.46
Work													
No	185 (42.6)	0.000	−0.002	Reference	0.000	−0.002	Reference	0.000	−0.002	Referencia	0.005	0.003	Referencia
Yes	249 (57.4)			0.93 (−0.84 to 0.92) β = 0.04			0.92 (−1.12 to 1.01) β = − 0.05			0.91 (−0.85 to 0.76) β = − 0.04			0.14 (− 1.32 to 0.19) β = − 0.56
Marital status													
Co-habitant	19 (4.38)	0.004	−0.003	Reference			Reference			Reference			Reference
Single	309 (71.20)			0.44 (−1.31 to 2.98) β = 0.84			0.97 (−2.64 to 2.54) β = −0.05			0.34 (−1.01 to 2.89) β = 0.95			0.80 (− 2.07 to 1.60) β = − 0.23
Married	98 (22.58)			0.77 (−1.94 to 2.61) β = 0.34	0.005	−0.003	0.76 (−2.32 to 3.18) β = 0.43	0.003	−0.004	0.46 (−1.28 to 2.85) β = 0.78	0.006	− 0.001	0.70 (− 2.32 to 1.56) β = − 0.38
Widower	8 (1.84)			0.95 (−3.94 to 3.71) β = − 0.11		|	0.33 (−6.90 to 2.35) β = − 2.62			0.97 (− 3.42 to 3.55) β = 0.06			0.12 (−5.61 to 0.92) β = − 2.34
Fear of coronavirus (family)													
No	124 (28.57)	0.005	0.002	Reference	0.003	0.001	Reference	0.001	−0.002	Reference	0.001	−0.001	Reference
Yes	310 (71.43)			0.16 (−1.66 to 0.27) β = − 0.69			0.27 (− 1.81 to 0.51) β = − 0.65			0.62 (− 1.09 to 0.66) β = − 0.22			0.53 (− 1.09 to 0.56) β = − 0.26
Product Concern													
Little or nothing	211 (48.62)	0.009	0.004	Reference	0.004	− 0.001	Reference	0.006	0.001	Reference	0.003	−0.002	Reference
Moderated	207 (47.70)			0.05*(−1.77 to − 0.001) **β = − 0.89**			0.38 (− 1.55 to 0.59) β = − 0.48			0.21 (− 1.32 to 0.29) β = − 0.51			0.25 (− 1.20 to 0.31) β = − 0.44
Severe	16 (3.69)			0.77 (− 2.69 to 2.004) β = − 0.34			0.30 (−4.35 to 1.33) β = − 1.51			0.22 (−3.47 to 0.81) β = − 1.33			0.69 (−2.41 to 1.61) β = − 0.40
Cause of concern													
Children and family care	44 (10.14)			Reference			Reference			Reference			Reference
Domestic work	18 (4.15)			0.69 (−3.05 to 2.04) β = − 0.51			0.38 (−1.69 to 4.45) β = 1.38			0.10 (−0.38 to 4.24) β = 1.93			0.72 (−1.77 to 2.58) β = 0.40
Social isolation	169 (38.94)	0.003	−0.009	0.82 (−1.73 to 1.36) β = − 0.18	0.009	−0.003	0.60 (− 1.31 to 2.41) β = 0.55	0.009	−0.003	0.48 (− 0.89 to 1.90) β = 0.50	0.005	−0.006	0.49 (− 0.85 to 1.78) β = 0.46
Not being able to work	121 (27.88)			0.73 (− 1.88 to 1.32) β = − 0.28			0.23 (−0.74 to 3.12) β = 1.19			0.84 (− 1.31 to 1.60) β = 0.15			0.68 (− 1.08 to 1.65) β = 0.29
Working without family	16 (3.69)			0.76 (−3.07 to 2.24) β = −0.42			0.86 (−3.48 to 2.93) β = − 0.28			0.61 (−1.78 to 3.04) β = 0.63			0.99 (−2.27 to 2.26) β = − 0.01
Teleworking	66 (15.21)			0.33 (−2.65 to 0.89) β = − 0.88			0.91 (− 2.27 to 2.01) β = − 0.13			0.91 (− 1.52 to 1.70) β = 0.09			0.68 (− 1.83 to 1.19) β = − 0.32

* p < 0.05; ** *p* < 0.01; *** *p* < 0.001

### General health status based on socio-economic profile **(**Table 1**)**

The sample adjusts to a normal distribution (±1.5 threshold) [46] where 40.80% (*n* = 177) of respondents reported psychological distress in contrast to non-cases (59.20%), with a cut-off point of 23/24 [36]. Men reported lower somatic and anxiety/insomnia symptom scores than women (β = − 1.87; β = − 1.91) respectively. The 59–68 age group has fewer somatic symptoms than younger age groups (β = − 2.09). Likewise, the 29–38-year-old group scored less in anxiety / insomnia (β = − 1.46) over the rest of the age groups. It is also observed that the groups from 49 to 58 years old (β = − 1.35) and 59 to 68 years old (β = − 3.11) score lower in social dysfunction than the younger age groups. With respect to severe depression, the 29–68 year-old group present lower scores (β = − 1.26 to − 2.36), with respect to the 18–28 year-old group (β = − 1.32). When observing educational levels, participants who have graduate (β = 13.16), undergraduate (β = 7.32) and university (β = 3.02) degrees present higher somatic symptoms than those who have high school. Similar tendency is presented in the anxiety / insomnia scales; graduate (β = 13.18), undergraduate (β = 6.45) and college (β = 1.61); in the social dysfunction scale: graduate (β = 6.98), undergraduate (β = 4.94) and college (β = 2.01); Also, in severe depression: graduate (β = 4.84), undergraduate (β = 3.02) and college (β = 0.16). Likewise, there are lower somatic symptoms (β = − 0.89), in participants who have moderate concern for the absence of hygiene products (protection, antibacterial gel, chinstraps and others) than those who do not worry about them.

### Association between sociodemographic variables and subscales of active and passive coping strategies toward the COVID-19 (Tables 2, 3 and 4)

Men are less likely than women to use positive reframing coping strategies (β = − 0.33). The 59–68 age group is identified as using less of the planning (β = − 0.81), positive reframing (β = − 1.14), and acceptance (β = − 0.96) coping strategies than the younger age group, similarly in the 39–48 age group (β = − 0.58) and the 49–58 age group (β = − 0.99), who are also less likely to use positive reframing strategy (β = − 0.66) and acceptance strategies (β = − 0.57). Looking at the level of study, participants with postgraduate degrees (β = − 0.53) and bachelor’s degrees (β = − 0.65) use the planning strategy less than participants with high school education, while participants with college education use positive reframing (β = 0.62) compared to those with high school education. We found that married participants use the planning coping strategy (β = − 0.79) and positive reframing (β = − 0.87) less than singles and cohabitants. It is evident that those who score moderate concern for the absence of hygiene products (protection, antibacterial gel, chinstraps, and others), are those most unlikely to use the positive reframing strategies (β = − 0.41) (see Tables 2 and 3).

**Table 2:** Descriptive analysis of the subscales of the Active and Passive Coping strategies

Active coping	*M*	*Mdn*	*Mo*	*DE*	*Min*	*Max*	*Q1*	*Q3*	CI(95%)
Active	3.77	4.00	4	1.45	0	6	3	5	(3.63, 3.91)
Planning	3.75	4.00	4	1.54	0	6	3	5	(3.61, 3.90)
Emotional support	2.66	2.50	2	1.63	0	6	2	4	(2.50, 2.82)
Social support	2.63	3.00	2	1.56	0	6	2	4	(2.48, 2.77)
Positive reframing	3.62	4.00	4	1.53	0	6	3	5	(3.48, 3.76)
Acceptance	4.18	4.00	4	1.39	0	6	3	5	(4.05, 4.31)
Humor	2.50	2.00	2	1.81	0	6	1	2	(2.32, 2.67)
Pasive coping									
Religion	2.83	3.00	2	1.87	0	6	1	4	(2.65, 3.01)
Denial	1.36	1.00	0	1.49	0	6	0	2	(1.22, 1.50)
Self-distraction	3.39	4.00	4	1.61	0	6	2	5	(3.24, 3.54)
Self-blame	1.93	2.00	2	1.43	0	6	1	3	(1.79, 2.08)
Disconnection	1.29	1.00	0	1.28	0	6	0	2	(1.17, 1.41)
Venting	2.01	2.00	2	1.34	0	6	1	3	(1.88, 2.13)
Substance use	.66	0.00	0	1.21	0	6	0	1	(0.55, 0.78)

**Table 3:** Association between sociodemographic variables and subscales of active coping strategies in covid-19

Variables	N(%)	Active			Planning			Positive reframing			Acceptance		
			Adjusted R-Squared	p (95% Confidence Interval)	R2	(AR2)	p(95% CI)	R2	(AR2)	p(95% CI)	R2	(AR2)	p(95% CI)
		R2	(AR2)	p(95% CI)									
Gender													
Woman	266 (61.3)	0.006	0.004	Reference	0.000	−0.002	Reference	0.011	0.001	Reference	0.008	0.006	Reference
Men	168 (38.7)			0.11(− 0.51 to 0.05) β = − 0.23			0.73 (− 0.25 to 0.35) β = 0.05			0.03*(− 0.63 to − 0.04) **β = − 0.33**			0.06 (− 0.53 to 0.01)
Age (years)													
18–28	181 (41.7)			Reference			Reference			Reference			Reference
29–38	124 (28.6)	0.009	−0.000	0.70 (− 0.40 to 0.27) β = − 0.06	0.026	0.017	0.51 (− 0.23 to 0.47) β = 0.12	0.063	0.054	0.23 (− 0.55 to 0.13) β = − 0.21			0.06 (− 0.62 to 0.01) β = − 0.30
39–48	51 (11.8)			0.97 (− 0.44 to 0.46) β = 0.01			0.19 (− 0.79 to 0.16) β = − 0.32			0.01*(− 1.05 to − 0.12) **β = − 0.58**	**0.043**	0.034	0.002*(− 1.09 to − 0.23) **β = − 0.66**
49–58	54 (12.4)			0.09 (−0.82 to 0.07) β = − 0.37			0.10 (− 0.85 to 0.08) β = − 0.39			0.001*(− 1.44 to − 0.53) **β = − 0.99**			0.01*(− 0.99 to − 0.16) **β = − 0.57**
59–68	24 (5.5)			0.26 (− 0.97 to 0.26) β = − 0.36			0.02*(− 1.46 to − 0.16) **β = − 0.81**			0.001*(− 1.78 to − 0.51) **β = − 1.14**			0.002*(− 1.50 to − 0.34) **β = − 0.96**
Education level													
High school	64 (14.75)			Reference			Reference			Reference			Reference
College	174 (40.09)	0.008	0.002	0.06 (− 0.01 to 0.82) β = 0.41	0.020	0.013	0.15 (− 0.76 to 0.12) β = − 0.32	0.027	0.020	0.01*(0.18 to 1.05) **β = 0.62**	0.013	0.006	0.80 (− 0.35 to 0.45) β = 0.05
Bachelor	116 (26.73)			0.27 (− 0.19 to 0.69) β = 0.25			0.01*(− 1.13 to − 0.18) **β = − 0.65**			0.63 (− 0.35 to 0.58) β = 0.11			0.48 (− 0.58 to 0.27) β = − 0.15
Postgrade	80 (18.43)			0.19 (− 0.16 to 0.79) β = 0.32			0.04*(− 1.04 to − 0.03) **β = − 0.53**			0.11 (− 0.09 to 0.91) β = 0.41			0.12 (− 0.82 to 0.09) β = − 0.37
Nationality													
Peruvian	409 (94.2)	0.003		Reference	0.005	0.003	Reference	0.000	−0.002	Reference	0.002	−0.000	Reference
Foreign	25 (5.8)		0.001	0.24 (−0.94 to 0.23) β = − 0.35			0.15 (−1.08 to 0.16) β = − 0.46			0.74 (− 0.72 to 0.52) β = − 0.11			0.34 (− 0.84 to 0.29) β = − 0.27
Work													
No	185 (42.6)	0.002	−0.000	Reference	0.001	−0.001	Reference	0.001	−0.001	Reference	0.003	0.000	Reference
Yes	249 (57.4)			0.34 (−0.41 to 0.14) β = − 0.13			0.55 (− 0.38 to 0.20) β = − 0.09			0.51 (− 0.38 to 0.19) β = − 0.09			0.29 (− 0.41 to 0.12) β = − 0.14
Marital status													
Co-habitant	19 (4.38)			Reference			Reference			Reference			Reference
Single	309 (71.20)	0.011	0.003	0.31 (−1.02 to 0.32) β = −0.35	0.012	0.005	0.16 (−1.23 to 0.19) β = − 0.52	0.016	0.009	0.09 (− 1.31 to 0.11) β = − 0.60	0.014	0.007	0.47 (− 0.88 to 0.40) β = − 0.24
Married	98 (22.58)			0.16 (− 1.23 to 0.19) β = − 0.52			0.04*(− 1.55 to − 0.04) **β = − 0.79**			0.02* (− 1.63 to − 0.12) **β = − 0.87**			0.09 (− 1.25 to 0.11) β = − 0.58
Widower	8 (1.84)			0.06 (−2.35 to 0.04) β = − 1.16			0.21 (−2.09 to 0.46) β = − 0.82			0.07 (− 2.40 to 0.13) β = − 1.14			0.80 (− 0.99 to 1.30) β = 0.15
Fear of coronavirus disease (family)													
No	124 (28.57)	0.003	−0.002	Reference	0.000	−0.002	Reference	0.000	−0.002	Reference	0.000	−0.002	Reference
Yes	310 (71.43)			0.73 (−0.25 to 0.36) β = 0.05			0.76 (−0.27 to 0.37) β = 0.05			0.99 (−0.32 to 0.32) β = − 0.001			0.76 (− 0.25 to 0.34) β = 0.05
Product Concern													
Little or nothing	211 (48.62)			Reference			Reference			Reference			Reference
Moderate	207 (47.70)	0.007	0.002	0.15 (−0.48 to 0.07) β = − 0.21	0.006	0.001	0.30 (− 0.45 to 0.14) β = − 0.16	0.020	0.016	0.01*(− 0.70 to − 0.12) **β = − 0.41**	0.012	0.007	0.05 (− 0.53 to 0.001) β = − 0.27
Severe	16 (3.69)			0.23 (−1.19 to 0.29) β = − 0.45			0.18 (− 1.32 to 0.25) β = − 0.54			0.09 (− 1.43 to 0.12) β = − 0.61			0.45 (− 0.44 to 0.97) β = 0.27
Causes of concern													
Children and family care	44 (10.14)			Reference			Reference			Reference			Reference
Domestic work	18 (4.15)	0.017	0.006	0.51 (−0.53 to 1.06) β = 0.27	0.017	0.006	0.81 (−0.95 to 0.74) β = − 0.10	0.011	− 0.001	0.68 (− 0.67 to 1.01) β = 0.17	0.194	0.008	0.08 (− 0.08 to 1.43) β = 0.68
Social isolation	169 (38.94)			1.09 (−0.80 to 0.16) β = − 0.32			0.43 (− 0.31 to 0.72) β = 0.20			0.60 (− 0.64 to 0.37) β = − 0.13			0.30 (− 0.22 to 0.69) β = 0.24
Not being able to work	121 (27.88)			0.11 (− 0.91 to 0.09) β = − 0.41			0.81 (− 0.59 to 0.47) β = − 0.06			0.30 (− 0.80 to 0.25) β = − 0.28			0.69 (− 0.38 to 0.57) β = 0.09
Working without family	16 (3.69)			0.05 (−1.65 to 0.01) β = − 0.82			0.24 (− 1.42 to 0.35) β = − 0.53			0.11 (− 1.58 to 0.16) β = − 0.71			0.17 (− 1.34 to 0.24) β = − 0.54
Teleworking	66 (15.21)			0.37 (− 0.80 to 0.30) β = − 0.25			0.22 (− 0.22 to 0.96) β = 0.37			0.91 (− 0.61 to 0.55) β = − 0.03			0.91 (− 0.49 to 0.56) β = 0.03

* p < 0.05; ** p < 0.01; *** p < 0.001

**Table 4:** Association between sociodemographic variables and subscales of passive coping during COVID-19

Variables	N(%)	Religion			Self-distraction			Self-blame			Venting		
		R-Squared	Adjusted R-Squared	p (95% Confidence Interval)	R2	(AR2)	p(95% CI)	R2	(AR2)	p(95% CI)	R2	(AR2)	p(95% CI)
		R2	(AR2)	p(95% CI)									
Gender													
Woman	266 (61.3)	0.049	0.047	Reference	0.010	0.008	Reference	0.007	0.004	Reference	0.016	0.014	Reference
Men	168 (38.7)			0.001*(−1.20 to − 0.49) **β = −0.84**			0.03*(− 0.64 a − 0.02) **β = − 0.33**			0.09 (− 0.04 to 0.51) β = 0.24			0.01*(− 0.60 to − 0.09) **β = − 0.35**
Age (year)													
18–28	181 (41.7)			Reference			Reference			Reference			Reference
29–38	124 (28.6)	0.021	0.012	0.57 (−0.30 to 0.54) β = 0.12	0.075	0.066	0.003*(−0.90 to − 0.18) **β = − 0.54**	0.038	0.029	0.02*(− 0.69 to − 0.05) **β = − 0.37**	0.015	0.006	0.12 (− 0.54 to 0.06) β = − 0.24
39–48	51 (11.8)			0.01*(0.16 to 1.31) **β = 0.74**			0.01*(− 1.12 to − 0.15) **β = − 0.64**			0.02*(− 0.93 to − 0.06) **β = − 0.50**			0.33 (− 0.62 to 0.21) β = − 0.21
49–58	54 (12.4)			0.13 (− 0.12 to 1.01) β = 0.44			0.001*(− 1.56 to − 0.61) **β = − 1.09**			0.001*(− 1.22 to − 0.37) **β = − 0.80**			0.25 (− 0.64 to 0.16) β = − 0.24
59–68	24 (5.5)			0.07 (− 0.07 to 1.51) β = 0.72			0.001*(− 2.08 to − 0.75) **β = − 1.42**			0.09 (− 1.11 to 0.08) β = − 0.52			0.02*(− 1.23 to − 0.09) **β = − 0.66**
Educational level													
High school				Reference			Reference			Reference			Reference
College	174 (40.09)	0.012	0.005	0.03*(0.07 to 1.14) **β = 0.61**	0.025	0.018	0.58 (− 0.33 to 0.58) β = 0.13	0.063	0.056	0.24 (− 0.16 to 0.63) β = 0.24	0.038	0.031	0.01*(0.12 to 0.88) **β = 0.50**
Bachelor	116 (26.73)			0.06 (− 0.02 to 1.11) β = 0.54			0.18 (−0.15 to 0.81) β = 0.33			0.003*(0.21 to 1.06) **β = 0.64**			0.002*(0.24 to 1.05) **β = 0.65**
Posgrade	80 (18.43)			0.09 (−0.09 to 1.13) β = 0.52			0.004*(0.23 to 1.29) **β = 0.76**			0.001*(0.61 to 1.52) **β = 1.07**			0.001*(0.44 to 1.31) **β = 0.88**
Nationality													
Peruvian	409 (94.2)	0.000	−0.002	Reference	0.000	−0.002	Reference	0.004	0.001	Reference	0.000	−0.002	Reference
Foreigner(living in Peru)	25 (5.8)			0.93 (−0.78 to 0.72) β = − 0.03			0.82 (− 0.72 to 0.57) β = − 0.07			0.21 (− 0.21 to 0.94) β = 0.37			0.97 (− 0.55 to 0.54) β = 0.01
Work													
No	185 (42.6)	0.000	−0.002	Reference	0.001	−0.001	Reference	0.001	−0.001	Reference	0.000	−0.002	Reference
Yes	249 (57.4)			0.81 (−0.31 to 0.39) β = 0.04			0.51 (−0.41 to 0.20) β = − 0.10			0.48 (− 0.37 to 0.17) β = − 0.09			0.90 (− 0.27 to 0.23) β = − 0.02
Marital status													
Co-habitant	19 (4.38)			Reference			Reference			Reference			Reference
Single	309 (71.20)	0.008	0.001	0.74 (−0.72 to 1.01) β = 0.14	0.004	−0.003	0.38 (−1.08 to 0.42) β = − 0.33	0.009	0.002	0.26 (− 0.28 to 1.03) β = 0.38	0.011	0.004	0.94 (− 0.59 to 0.64) β = 0.02
Married	98 (22.58)			0.59 (−1.16 to 0.67) β = − 0.25			0.27 (− 1.23 to 0.35) β = − 0.44			0.24 (− 0.28 to 1.12) β = 0.42			0.37 (− 0.95 to 0.36) β = − 0.30
Widower	8 (1.84)			0.71 (− 1.83 to 1.25) β = − 0.29			0.27 (−2.07 to 0.60) β = − 0.74			0.45 (− 1.63 to 0.72) β = − 0.45			0.56 (− 0.78 to 1.42) β = 0.32
Fear of contracting coronavirus													
No	124 (28.57)	0.000	− 0.002	Reference	0.003	0.000	Reference	0.000	−0.002	Reference	0.000	−0.002	Reference
Sí	310 (71.43)			0.78 (−0.33 to 0.44) β = 0.05			0.25 (−0.14 to 0.53) β = 0.20			0.92 (−0.31 to 0.28) β = − 0.01			0.88 (− 0.25 to 0.30) β = 0.02
Fear of products													
Little or nothing	211 (48.62)			Reference			Reference			Reference			Reference
Moderate	207 (47.70)	0.002	−0.002	0.64 (−0.44 to 0.27) β = − 0.08	0.004	−0.000	0.42 (− 0.43 to 0.18) β = − 0.13	0.003	−0.002	0.55 (− 0.35 to 0.19) β = − 0.08	0.009	0.004	0.10 (− 0.47 to 0.04) β = − 0.22
Severe	16 (3.69)			0.35 (−1.40 to 0.50) β = − 0.45			0.20 (−1.35 to 0.28) β = − 0.53			0.33 (− 1.08 to 0.36) β = − 0.36			0.14 (− 1.18 to 0.17) β = − 0.50
Causes of concern													
Children and family care	44 (10.14)			Reference			Reference			Reference			Reference
Domestic work	18 (4.15)	0.009	−0.002	0.56 (−1.33 to 0.72) β = − 0.30	0.007	−0.004	0.94 (− 0.85 to 0.92) β = 0.03	0.008	−0.003	0.29 (− 1.21 to 0.36) β = − 0.42	0.014	0.002	0.51 (− 0.97 to 0.49) β = − 0.24
Social isolation	169 (38.94)			0.28 (− 0.94 to 0.28) β = − 0.34			0.87 (− 0.58 to 0.49) β = − 0.04			0.98 (− 0.47 to 0.48) β = 0.01			0.74 (− 0.37 to 0.51) β = 0.07
Not being able to work	121 (27.88)			0.12 (− 1.16 to 0.13) β = − 0.52			0.34 (− 0.82 to 0.29) β = − 0.27			0.73 (− 0.57 to 0.41) β = − 0.08			0.59 (−0.33 to 0.58) β = 0.12
Working without family	16 (3.69)			0.74 (−0.89 to 1.25) β = 0.18			0.21 (− 1.51 to 0.34) β = − 0.59			0.25 (−1.29 to 0.34) β = − 0.48			0.37 (− 1.11 to 0.42) β = − 0.35
Teleworking	66 (15.21)			0.64 (− 0.88 to 0.54) β = − 0.17			0.64 (− 0.76 to 0.47) β = − 0.14			0.76 (− 0.46 to 0.63) β = 0.08			0.15 (− 0.13 to 0.88) β = 0.38

* *p* < 0.05; ** *p* < 0.01; *** p < 0.001

Further statistical test regarding passive coping strategies (Table 4) revealed that, men are less likely to use religion (β = − 0.84), self-distraction (β = − 0.33), and venting (β = − 0.35) compared to women in the studied population. The 39–48 age group employs more religion-base responses (β = 0.74) than younger age groups. Regarding self-distraction behavior, it occurs to a lesser extent as age increases, i.e., for those in the age group 29 to 38 years (β = − 0.54); 39 to 48 years (β = − 0.64); 49 to 58 years (β = − 1.09) and 59 to 68 years (β = − 1.42). Similarly, is the case for the self-blaming strategy in all age categories: 29–38 age group (β = − 0.37), 39–48 age group (β = − 0.50), 49–58 age group (β = − 0.80). The older group (59–68 years old) uses the venting strategy to a lesser extent compared to the younger age groups. It should be noted that, while college students use more religion-based (β = 0.61) and venting (β = 0.50) strategies, bachelor’s degree students use self-blaming behaviors (β = 0.64); In contrast to professionals with graduate degrees who more likely use self-distraction (β = 0.76), self-blame (β = 1.07), and venting (β = 0.88) as passive coping strategies compared to high-school going participants in the studied population. As shown in Table 5.

**Table 5:**
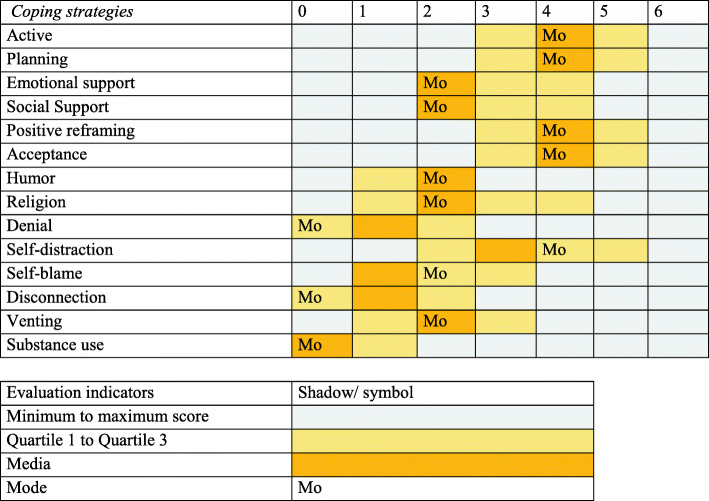
Assessment Indicators Active and Passive Coping Strategies

### Pearson’s correlations between psychological health and coping strategies **(**Table 6**)**

Table 6 depicts health indicators. The positive correlation suggests an increase in somatic symptoms (r = 0.20**), anxiety (r = 0.13**) and social dysfunction (r = 0.15**) among those with better strategy of emotional support. Moreover, greater anxiety/insomnia (r = 0.16**) and social dysfunction (r = 0.13**) among those with higher social support. The planning strategy correlates inversely with severe depression (r = − 0.19**). The higher is the situation-acceptance strategy, the lower the indicators of somatic symptoms (r = − 0.10*), anxiety/insomnia (r = − 0.10*), and severe depression (r = − 0.11*) (*p < 0.05) among the respondents. Finally, active strategies of positive reframing and humor do not correlate with any indicator measured by the General Health Scale (GHQ).

**Table 6:** Pearson’s correlations for General Health indicators (GHQ)and active and passive coping strategies (COPE)

	1	2	3	4	5	6	7	8	9	10	11	12	13	14	15	16	17	18
1. Somatic symptoms	1																	
2. Anxiety/insomnia	.69^**^	1																
3. Social dysfunction	.55^**^	.56^**^	1															
4. Severe depression	.49^**^	.54^**^	.50^**^	1														
5. Active	.03	.03	.02	−.12^*^	1													
6. Planning	−.08	−.06	−.02	−.19^**^	.66^**^	1												
7. Emotional support	.20^**^	.13^**^	.15^**^	.01	.32^**^	.28^**^	1											
8. Social support	.12^*^	.16^**^	.13^**^	.02	.34^**^	.38^**^	.54^**^	1										
9. Positive reframing	−.02	.01	.05	−.10	.54^**^	.51^**^	.33^**^	.29^**^	1									
10. Acceptance	−.10^*^	−.10^*^	.02	−.11^*^	.52^**^	.53^**^	.15^**^	.28^**^	.56^**^	1								
11. Humor	.05	−.01	.09	.04	.27^**^	.36^**^	.17^**^	.29^**^	.34^**^	.38^**^	1							
12. Religion	.04	.05	.03	−.10	.35^**^	.34^**^	.39^**^	.32^**^	.34^**^	.29^**^	0	1						
13. Denial	.21^**^	.23^**^	.17^**^	.18^**^	−.07	.04	.16^**^	.26^**^	−.14^**^	−.10^*^	.10^*^	.12^*^	1					
14. Self-distraction	.15^**^	.27^**^	.26^**^	.15^**^	.43^**^	.48^**^	.29^**^	.39^**^	.50^**^	.45^**^	.34^**^	.31^**^	.04	1				
15. Self-blame	.23^**^	.26^**^	.29^**^	.41^**^	.21^**^	.18^**^	.17^**^	.24^**^	.16^**^	.10^*^	.35^**^	.02	.22^**^	.32^**^	1			
16. Disengagement	.23^**^	.23^**^	.36^**^	.36^**^	−.05	−.04	.18^**^	.09	.05	−.04	.16^**^	.05	.23^**^	.12^*^	.33^**^	1		
17. Venting	.16^**^	.15^**^	.20^**^	.19^**^	.32^**^	.29^**^	.32^**^	.32^**^	.25^**^	.20^**^	.28^**^	.19^**^	.14^**^	.34^**^	.38^**^	.22^**^	1	
18. Substance use	.20^**^	.15^**^	.20^**^	.21^**^	−.07	−.11^*^	.09	.06	−.15^**^	−.08	.12^*^	.01	.32^**^	.01	.22^**^	.19^**^	.15^**^	1

* p < 0.05; ** p < 0.01

### Path analysis results related to active and passive coping strategies **(**Figs. 1 and 2**)**

**Fig. 1:**
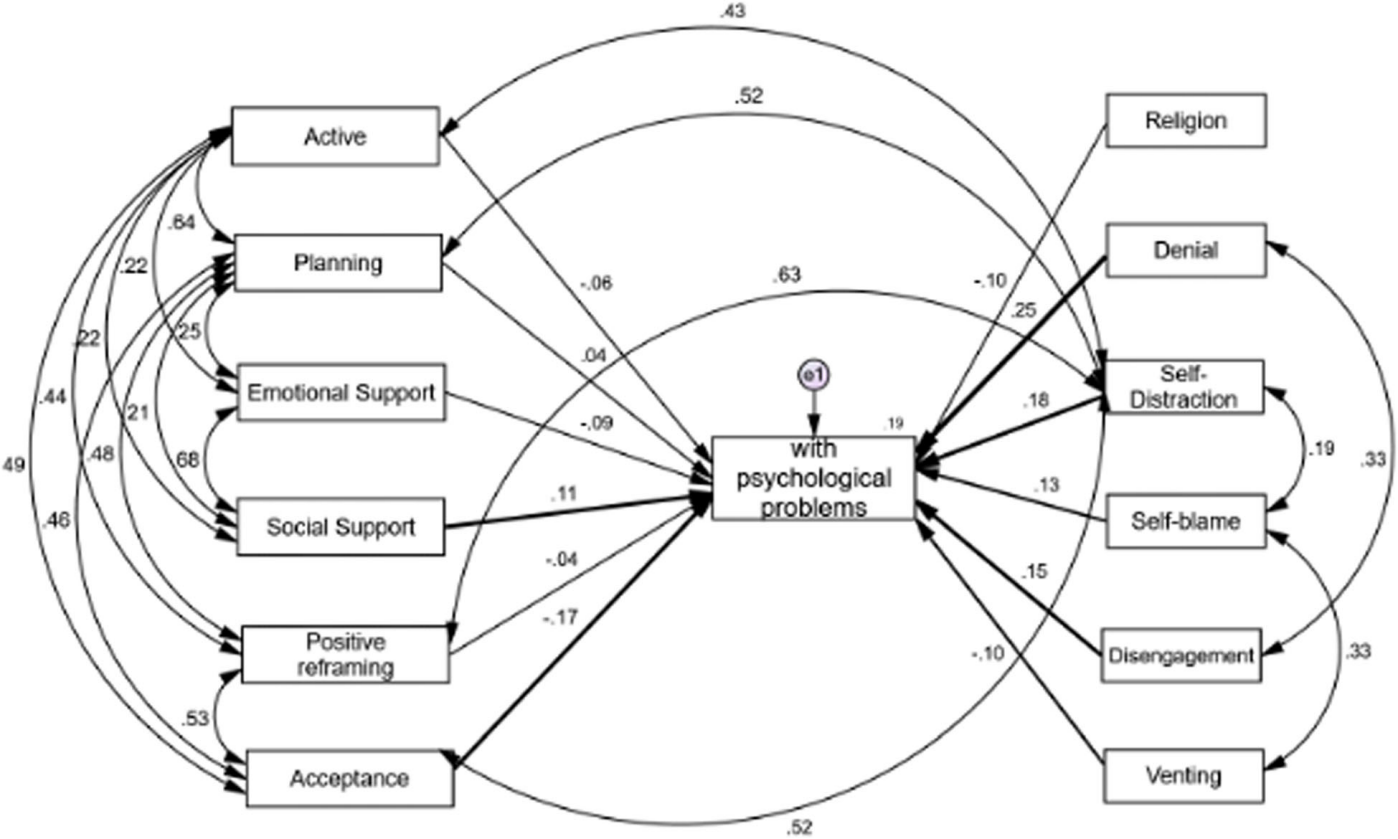
Report of the path analysis. Standardized coefficients of active and passive coping strategies in participants with psychological problems (N = 177; *p < .05)

**Fig. 2:**
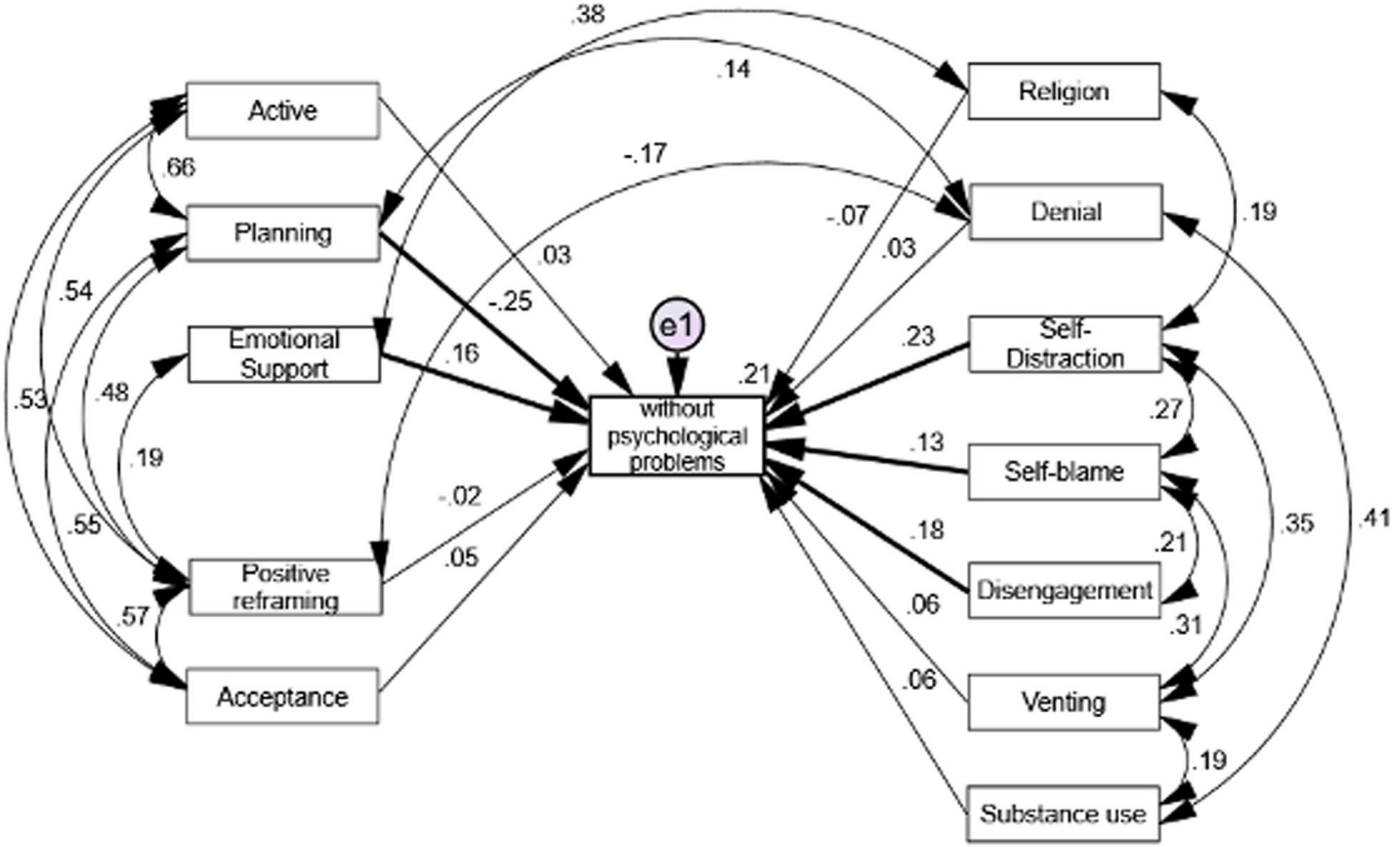
Report of the path analysis. Standardized coefficients of active and passive coping strategies in participants without psychological problems (*N* = 257; *p < .05)

Results derived from standardized path analysis coefficients shows active and passive responses (Figs. 1 and 2). As such, social acceptance and support-seeking behaviors are active strategies, which are more likely to be used by individuals who rated with psychological distress. The model retrieved acceptable goodness-of-fit indexes (X^2^/gl = 3.09; GFI = 0.880; IFC = 0.85 and RMSEA = 0.10 (IC90% 0.08, 0. 12). Although it is a model that does not strictly meet the expected parameters, the values are close and indicate that the active strategies used by participants with psychological distress are acceptance (negative), social support (positive); the passive strategies such as denial (positive way), self-distraction (positive), self-blaming (positive), disconnection (positive) and venting (negative). Except humor (active strategy) and substance use (passive strategy) that do not support the explanation. The model explains 19% of the variance (R^2^ = 0.19) on the impact of the active and passive coping strategies among patients with psychological distress, with adequate adjustment index. The strategies that do not support the model are humor (active strategy) and substance use (passive strategy). (Fig. 1).

On the contrary Fig. 2 describes coefficients for active and passive strategies for participants who reported absence of psychological distress. For one hand, the model has acceptable goodness-of-fit indices (X^2^/gl = 5.73; GFI = 0.86; CAI = 0.75 and RMSEA = 0.11 / IC90% 0.09, 0. 139), with close values to the expected parameters, indicating that active strategies among participants without psychological distress are: emotional support (positive) and planning (positive). For the other hand, the passive strategies people without mental illness reported are self-distraction (positive), self-blame (positive), and disconnection (positive). Particularly, the active strategies that do not support the model are humor and social support. This model (R^2^ = 0. 21) explain 21% of the variance, that is that 21% of the participants who do not present psychological distress use the aforementioned coping strategies, with adequate adjustment indexes.

## Discussion

As expected, our findings suggest that throughout the period of COVID-19 social isolation (April–May), during which this survey was conducted, respondents experienced psychosomatic symptoms, anxiety, social dysfunction, and severe depression as assessed by the self-reported GHQ-28 questionnaire. Gender, age, education level, and having moderate concerns about access to sanitization products were associated with mental health distress. Other factors that include nationality, employment, marital status, and whether one is afraid of the coronavirus disease were not significantly associated with the presence of psychological symptoms. Similar to previous research performed at the beginning of the pandemic in China, marital and parental status were not associated with mental health excepting employment which was linked with lower stress and anxiety [12].

One of the most striking results to emerge from this study is that age and gender are associated to psychological distress. Regarding psychological manifestations, it was observed that people are less likely to suffer from major depression as age increases. Men scored lower levels of somatic symptoms, and anxiety/insomnia compared to women. Moreover, the regression model demonstrated participants with higher education scored greater in somatic symptoms (R^2=^0.87) during the COVID-19 lockdown. We firstly hypothesized that somatic symptom and higher education association may be explained as an interactive effect related to gender (R^2=^0.03) given that our sample is unintentionally mostly composed by women (61.30%), and being this point supported by prior studies which had consistently noted worse somatic symptoms [47], anxiety, and depression amongst women population [48]. Another possible explanation might be related to subjective mindset and beliefs, where perceived risk of stress triggers increased physiological disfunction [20]. Education level alone, without attention to local practices and beliefs is insufficient to understand the mental health impact of a pandemic [49]. Therefore, how people make appraisal of external situations should not be ignored.

Particularly, the shift in working conditions and its virtual infrastructure encouraged varied industries in Peru to implement teleworking for the first time [50]. Educated individuals might highly likely experience greater cognitive demand in the face of tremendous adversity, it is reasonable thereof higher somatic symptoms also associated to economic and social conditions [51] in educated participants. However, this result may reflect a temporary somatic reaction to the onset of the pandemic.

Peruvians have experienced a loss or disruption of employment, financial hardships, as well as experiencing scarcity of basic provisions which may affect their health status. The studied population reported concern about social isolation (38.94%), not being able to work (27.88%), changes in circumstances including working without family (3.69%), doing domestic work (4.15%), caring for children and family (10.14%). In contrast to earlier studies which had suggested higher rates of anxiety associated to sense of concern for themselves and their families [52]. It could be argued that results might be partly related to the high number of young, employed respondents in our study. Acknowledging that the Peruvian population is largely nuclear households (53.9%) including couple with or without children, followed by extended families (20.6%) and single person households (16.8%) [53]. Concerns in the majority of single respondents (71.2%) were less likely to include others. Finally, shortage of basic provisions and increased spending on sanitizers was ranked as a cause of distresses linked to moderate somatic symptoms in nearly half of respondent (47.70%), use of tonics and medicines to not get sick or prevent physical discomfort also reported on similar studies [7].

On studying the association between socio-demographic characteristics and coping strategies we identified significant relationship between being women, younger groups (< 39-year-old), college students, and being single respondents tend to use more active coping strategies. Active coping recounted as planning, positive reframing, and acceptance. On the other hand, passive coping strategies such as self-distraction and self-incrimination are less likely to be used as the age increases.

### Psychological problems and use of coping strategies

Contrary to expectations, we did not find a significant difference between people using active and passive coping strategies. Initially, we thought that active coping was typically used among people experiencing absence of mental distress. Surprisingly, both passive and active had been identified, regardless of psychological status among the studied population.

Controversy remains regarding stress-coping behavior when responding to unknown stressors [30]. Although, on the one hand, people with psychological distress are found to be more likely to use passive mechanisms to reduce emotional stress (i.e., through behaviors such as denial, self-distraction, self-blaming and behavioral disengagement), they also score high on active behavioral coping, (i.e., that they actively confront to emotional tension, through low acceptance of the situation, and high social support seeking). On the other hand, those participants without psychological distress were more likely to use active coping that included social support, followed by passive forms such as self-distraction, disconnection from activities, and planning. Further, studies made by Petzold’s indicate that acceptance of anxiety and negative emotions seems to be supportive passive strategies [54] to maintain psychological equilibrium. In this regard, people scoring high only in emotion-based response are more likely to report psychiatric symptoms, in contrast to those using both active problem-resolution and emotion management [9].

In our findings, the use of coping strategies is correlated with gender. We found that men use positive reframing strategies to a lesser extent than women. Students-based research described opposite results, where young men are more likely to use positive management responses (positive reframing and planning in stressful situations) [55]. The reason for this is not clear but it may have something to do with the ongoing pandemic, participants responded to the questionnaire under an unusual circumstance that may had influenced or distorted their self-perception in the face of the uncertainty [25, 56] and their ability to analyze and solve the problems [57]. Contrastingly, Asian-based studies reported the deployment of active styles focusing on active problem solving (active, social support and planning), which may significantly predict responses of anxiety (3.40%), anger (2.20%) and sadness (0.90%). Particularly, first-liner women workers may be more likely to use proactive, problem-centered coping in the face of the pandemic and less likely to use passive strategies than men [13]. This has some minor fluctuations in North America, where women are more likely to report strategies that focus on passive behaviors such as distraction, religion, and less humor [58].

Interestingly, there seems to be a relationship between religion-related coping beliefs and gender. The great deal of female respondent in our study (61.30%) informed using religion-based strategies probably to mitigate stress, being this explained by the Peruvian population identified as Catholic (nearly 76%) [53], compared to progressive countries. Also in line with recent Chinese studies in social networks, where most of women used belief-based responses during the pandemic [9].

Another passive coping style is related to self-distraction, which is less likely to occur as age increases (> 28 years old). It is noteworthy mentioning that whilst passive coping contributes on lessening powerlessness in the face of stress, it may also operate as a maladaptive strategy leading to psychological distress [5]. We found that the use of passive responses (denial, self-distraction, self-incrimination, venting, and religion) may reflect the unprecedented impact of the COVID-19 pandemic; that metaphorically it is understood as a chain of misadjusted responses that begins by rejecting the deadly consequences of the disease, not accepting reality, resorting then to activities to avoid thinking about the crisis and confronting the problem. Evidently, this study captures the nature of human perceptions in contexts of uncertainty. Individual variance in analyzing an addressing demanding life situations serves a moderating agent [30], particularly for mental health outcomes. These findings are of critical importance for developing and/or strengthening active and passive coping modalities to educate by gender, age, and level of education in the general population.

Although our survey was conducted two weeks after emergency was declared by the Peruvian government and being our study one of the first to investigate the impact of COVID-19 on mental health and coping strategies in the face of the crisis in Peru. Additional research is urged to monitor participants in the aftermath of the pandemic. Particularly, given the long-term need for severe restrictions and the impact on economic activity, and to examine the evolution of psychological distress after the state of emergency. Other studies on low-income populations with poor internet connection and restricted health access may provide further insight into coping strategies and the effects on mental health.

## Conclusions

As the pandemic is now persisting into a second year, there is still a compelling need to minimize the impact of the epidemic until vaccines become predominant and this includes a greater response to the mental health needs of the population. This report found moderate levels of psychological distress, greater issues regarding mental distress were associated to women, those with higher education, across all age groups, except the youngest (18 to 28 years). Peruvians would still benefit from appropriate interventions to address the mental health disturbances that have arisen during the pandemic. Policies are urged to support awareness and education toward active coping strategies. Our findings on the use of coping strategies also inform development of timely intervention programs to address long-term disorders arising during quarantine.

The authors acknowledge some limitations. First, there may be some selection bias, considering that only people with Internet access and/or knowledge of social networks, where the research was advertised, participated in the study. Second, although, the Internet-based survey method prevented possible coronavirus from spreading to researchers, the procedure excluded participants without computer or a cell phone. Our sample, thus, underrepresents those with low incomes and who did not use information technologies during the COVID-19 pandemic.

## Data Availability

﻿Materials, including data files can be obtained directly from the corresponding authors on request.

## Abbreviations

GHQ-28: The General Health Questionnaire
COPE-28: Coping Strategy Questionnaire
COVID-19: Coronavirus disease 2019 or 2019-nCoV, new virus linked to the same family of viruses as Severe Acute Respiratory Syndrome (SARS)
CAI: Comparative Adjustment Index
RMSEA: Root Mean Square Error of Approach
SEM: Structural Equation Models
GFI: Goodness-of-Fit Index
CI: Confidence interval

## References

[1] WHO (2020). Coronavirus Disease 2019 (COVID-19) Situation Reports. 2020 [Internet]. Vol. 2019, WHO Situation report.

[2] Adhikari SP, Meng S, Wu Y, Mao Y, Ye R, Wang Q (2020). Epidemiology , causes , clinical manifestation and diagnosis , prevention and control of coronavirus disease ( COVID-19 ) during the early outbreak period : a scoping review.

[3] Sohrabi C, Alsafi Z, O’Neill N, Khan M, Kerwan A, Al-Jabir A (2020). World Health Organization declares global emergency: a review of the 2019 novel coronavirus (COVID-19). Int J Surg.

[4] Mao Y, Lin W, Wen J, Chen G (2020). Clinical and pathological characteristics of 2019 novel coronavirus disease ( COVID-19 ): a systematic reviews.

[5] Sim K, Huak Chan Y, Chong PN, Chua HC, Wen SS (2010). Psychosocial and coping responses within the community health care setting towards a national outbreak of an infectious disease. J Psychosom Res.

[6] Torales J, O’Higgins M, Castaldelli-Maia JM, Ventriglio A. The outbreak of COVID-19 coronavirus and its impact on global mental health. Int J Soc Psychiatry. 2020;66(4):317–20. 10.1177/0020764020915212 32233719

[7] Öğütlü H. Turkey’s response to COVID-19 in terms of mental health. Ir J Psychol Med. 2020;37(3):222–5. 10.1017/ipm.2020.57 PMC7276500 32434621

[8] Lu W, Wang H, Lin Y, Li L (2020). Psychological status of medical workforce during the COVID-19 pandemic: a cross-sectional study. Psychiatry Res.

[9] Li S, Wang Y, Xue J, Zhao N, Zhu T. The Impact of COVID-19 Epidemic Declaration on Psychological Consequences: A Study on Active Weibo Users. Int J Environ Res Public Health. 2020;17(6):2032. 10.3390/ijerph17062032 PMC7143846 32204411

[10] Wang Y, Wang Y, Chen Y, Qin Q. Unique epidemiological and clinical features of the emerging 2019 novel coronavirus pneumonia (COVID-19) implicate special control measures. J Med Virol. 2020;92(6):568–76. 10.1002/jmv.25748 PMC7228347 32134116

[11] Qiu J, Shen B, Zhao M, Wang Z, Xie B, Xu Y (2020). A nationwide survey of psychological distress among Chinese people in the COVID-19 epidemic : implications and policy recommendations.

[12] Wang C, Pan R, Wan X, Tan Y, Xu L, Ho CS, et al. Immediate psychological responses and associated factors during the initial stage of the 2019 coronavirus disease (COVID-19) epidemic among the general population in China. Int J Environ Res Public Health. 2020;17(5):1729. 10.3390/ijerph17051729 PMC7084952 32155789

[13] Huang L, Xu FM, Liu HR. Emotional responses and coping strategies of nurses and nursing college students during COVID-19 outbreak. medRxiv. 2020; 10.1101/2020.03.05.20031898. 10.1371/journal.pone.0237303 PMC7413410 32764825

[14] Newby J, O’Moore K, Tang S, Christensen H, Faasse K. Acute mental health responses during the COVID-19 pandemic in Australia. PLOS ONE. 2020;15(7):e0236562. 10.1371/journal.pone.0236562 PMC7386645 32722711

[15] Weisman LS. COVID-19. World Health Organization-strategy update 2020.

[16] Brooks SK, Webster RK, Smith LE, Woodland L, Wessely S, Greenberg N, Rubin GJ (2020). The psychological impact of quarantine and how to reduce it: rapid review of the evidence. Lancet.

[17] Lazarus RS, Folkman S (1984). Stress, appraisal, and coping [internet]. Springer Publishing Company.

[18] Park CL, Adler NE (2003). Coping style as a predictor of health and well-being across the first year of medical school. Health Psychol.

[19] Smith BW, Kay VS, Hoyt TV, Bernard ML (2009). Predicting the anticipated emotional and behavioral responses to an avian flu outbreak. Am J Infect Control.

[20] Van Bavel JJ, Baicker K, Boggio PS, Capraro V, Cichocka A, Cikara M (2020). COVID-19 pandemic response. Nat Hum Behav.

[21] Ni JJ, Bordoloi S, Shao W, Garg A. COVID-19 and mental health: a review of the existing literature. Sci Total Environ. 2019;136486 Available from: https://doi.org/10.1016/j.scitotenv.2019.136486.

[22] Taylor S. The psychology of pandemics: preparing for the next global outbreak of infectious disease [internet]: Cambridge Scholars Publishing; 2019. Available from: https://books.google.com.pe/books?id=8mq1DwAAQBAJ

[23] Zhang SX, Wang Y, Rauch A, Wei F (2020). Health, distress and life satisfaction of people in China one month into the COVID-19 outbreak. SSRN Electron J.

[24] Barbisch D, Koenig KL, Shih FY (2015). Is there a case for quarantine? Perspectives from SARS to Ebola. Disaster Med Public Health Prep.

[25] Asmundson G, Taylor S. Coronaphobia: Fear and the 2019-nCoV outbreak. J Anxiety Disord. 2020;70:102196. 10.1016/j.janxdis.2020.102196 PMC7134790 32078967

[26] Weiss DS (1978). The Impact of Event Scale : Revised.

[27] Bali S, Stewart KA, Pate MA (2016). Long shadow of fear in an epidemic: Fearonomic effects of Ebola on the private sector in Nigeria. BMJ Glob Heal.

[28] Davis M, Stephenson N, Flowers P (2011). Compliant, complacent or panicked? Investigating the problematisation of the Australian general public in pandemic influenza control. Soc Sci Med.

[29] De Prensa N. Plataforma digital del Estado Peruano. Ministerio de Salud; 2020:15–7. [Internet]. [20 Apr 2020]. Retrieved from: https://covid19.minsa.gob.pe/.

[30] Billings AG, Moos RH (1981). The role of coping responses and social resources in attenuating the stress of life events. J Behav Med.

[31] Goldberg D, Williams P (1996). Cuestionario de Salud General (GHQ-28). Masson.

[32] De Arévalo F (2004). Assessment of the factor structure and reliability of the 28 item version of the general health questionnaire (GHQ-28) in El Salvador. Int J Clin Heal Psychol.

[33] Retolaza A, Mostajo A, de la Rica JR, Diaz A, Perez J, Aramberri I (1993). Validación del Cuestionario de Salud General de Goldberg (versión 28 ítems) en consultas de atención primaria. Rev Asoc Esp Neuropsiq.

[34] Vallejo MA, Rivera J, Esteve-vives J, Rodríguez-Muñoz M (2014). grupo ICAF. El cuestionario general de salud (GHQ-28) en pacientes con fibromialgia: propiedades psicométricas y adecuación. Clínica y Salud.

[35] Luis J, Leon V (2017). El coeficiente Omega: un método alternativo para la estimación de la confiabilidad. Rev Latinoam Ciencias Soc Niñez y Juv.

[36] Swallow BL, Lindow SW, Masson EA, Hay DM (2003). The use of the general health questionnaire (GHQ-28) to estimate prevalence of psychiatric disorder in early pregnancy. Psychol Heal Med.

[37] Morán C, Landero R, González MCT (2010). COPE-28: Un análisis psicométrico de la versión en Español del brief COPE. Univ Psychol.

[38] Carver CS (1997). You want to measure coping but your protocol’s too long: consider the brief COPE. Int J Behav Med.

[39] Paola S, Paola G, Judith G, Herrera-olaya GP, Sepúlveda-carrillo GJ (2010). Confiabilidad del cuestionario Brief COPE Inventory en versión en español para evaluar estrategias de afrontamiento en pacientes con cáncer de seno. Investig en Enfermería Imagen y Desarro.

[40] Huamaní Domínguez JA, Paredes Ayala AC (2018). Estrés y estrategias de afrontamiento: Estudio en familiares de pacientes con cáncer en la ciudad de Lima, 2018.

[41] StataCorp (2017). Stata Statistical Software: Released 15. Coll Station TX StataCorp LLC.

[42] Podsakoff PM, MacKenzie SB, Lee JY, Podsakoff NP (2003). Common method biases in behavioral research: a critical review of the literature and recommended remedies. J Appl Psychol.

[43] Bentler PM (1990). Comparative fit indexes in structural models. Psychol Bull.

[44] MacCallum RC, Browne MW, Sugawara HM (1996). Power analysis and determination of sample size for covariance structure modeling. Psychol Methods.

[45] Hu LT, Bentler PM (1999). Cutoff criteria for fit indexes in covariance structure analysis: conventional criteria versus new alternatives. Struct Equ Model.

[46] George D, Mallery M (2003). Using SPSS for Windows step by step: a simple guide and reference.

[47] Barari S, Caria S, Davola A, Ivchenko A, Jachimowicz J, King G (2020). Evaluating COVID-19 Public Health Messaging in Italy : Self-Reported Compliance and Growing Mental Health Concerns. medRxiv [working paper, not yet peer reviewed].

[48] Eliasen M, Kreiner S, Ebstrup JF, Poulsen CH, Lau CJ, Skovbjerg S (2016). Somatic symptoms: prevalence, co-occurrence and associations with self-perceived health and limitations due to physical health - a danish population-based study. PLoS One.

[49] Taylor S. The psychology of pandemics: Preparing for the next global outbreak of infectious disease: Cambridge Scholars Publishing; 2019

[50] Instituto Nacional de Salud y Centro Nacional de Epidemiología P y C de enfermedades-M (2020). COVID-19 en el Perú. Ministerio de Salud del Perú.

[51] Buitrago Ramírez F, Ciurana Misol R, Tizón JL, Fernández Alonso M del C (2021). COVID-19 pandemic and mental health: initial considerations from spanish primary health care. Aten Primaria.

[52] Erceg N, Ružojčić M, Galić Z (2020). Misbehaving in the Corona Crisis: The Role of Anxiety and Unfounded Beliefs.

[53] Instituto Nacional de Estadística e Informatica (2017). Censos Nacionales 2017: XII de Población, VII de Vivienda y III de Comunidades Indígenas.

[54] Petzold MB, Bendau A, Plag J, Pyrkosch L, Mascarell Maricic L, Betzler F (2020). Risk, resilience, psychological distress, and anxiety at the beginning of the COVID-19 pandemic in Germany. Brain Behav.

[55] Cabanach RG, Fariña F, Freire C, González P, Ferradás M del M (2015). Diferencias en el afrontamiento del estrés en estudiantes universitarios hombres y mujeres. Eur J Educ Psychol.

[56] Kang L, Ma S, Chen M, Yang J, Wang Y, Li R, et al. Impact on mental health and perceptions of psychological care among medical and nursing staff in Wuhan during the 2019 novel coronavirus disease outbreak: A cross-sectional study. Brain Behav Immun. 2020;(March):1–7. 10.1016/j.bbi.2020.03.028 PMC7118532 32240764

[57] Yang Y, Merrill EC (2017). Cognitive and personality characteristics of masculinity and femininity predict wayfinding competence and strategies of men and women. Sex Roles.

[58] Park CL, Russell BS, Fendrich M, Finkelstein-Fox L, Hutchison M, Becker J (2020). Americans’ COVID-19 stress, coping, and adherence to CDC guidelines. J Gen Intern Med.

